# Towards a mitogenomic phylogeny of mud dragons (Kinorhyncha): two new mitogenomes from the Pycnophyidae family

**DOI:** 10.1038/s41598-026-54168-x

**Published:** 2026-05-23

**Authors:** Marek Lubośny, Aleksandra Zalewska, Maria Herranz, Martin V. Sørensen, Katarzyna Grzelak

**Affiliations:** 1 Department of Genetics and Marine Biotechnology, Institute of Oceanology, Polish Academy of Sciences, Sopot, Poland; 2 Laboratory of Molecular Evolution and Bioinformatics, Department of Evolutionary Genetics and Biosystematics, Faculty of Biology, University of Gdańsk, Gdańsk, Poland; 3 Department of Marine Ecology, Institute of Oceanology, Polish Academy of Sciences, Sopot, Poland; 4 Department of Biology, Rey Juan Carlos University (URJC), Móstoles, Spain; 5 Global Change Research Institute (IICG-URJC), Móstoles, Spain; 6 Natural History Museum of Denmark, University of Copenhagen, Copenhagen, Denmark

**Keywords:** mtDNA, Lack of stop codon, Mud dragons, Phylogenetic, Pycnophyidae, Ecology, Ecology, Evolution, Genetics, Zoology

## Abstract

Mud dragons (Kinorhyncha) are an understudied phylum of microscopic marine invertebrates inhabiting a wide range of marine sediments, from shallow coastal habitats to abyssal depths, and occurring from tropical to polar regions. Despite their broad distribution, their minute size often makes them difficult to study. Consequently, molecular resources for the group remain extremely limited and only three complete mitogenomes have been published from this phylum to date. To help fill this substantial gap in genetic data, we sequenced and annotated two new complete mitogenomes from the family Pycnophyidae, *Pycnophyes greenlandicus* Higgins & Kristensen, 1988 and *Cristaphyes cryopygus* (Higgins & Kristensen, 1988). Obtained mtDNA sequences were compared with available transcriptomic data from other Kinorhyncha species, providing a more comprehensive basis for the phylogenetic analysis of the phylum. The results showcased unexpected rearrangements in the gene order across all examined taxa, an unusual *cox1*–tRNA-Glu genes overlap and an unstable phylogenetic position within the Pycnophyidae family. These new sequences not only significantly expand the mitogenomic data available for Kinorhyncha, but also provide an important step toward a better understanding of phylogenetic relationships within the phylum.

**Supplementary Information:**

The online version contains supplementary material available at 10.1038/s41598-026-54168-x.

## Introduction

Animal mitochondrial DNA (mtDNA) is generally described as a small (~ 16k base pairs) circular molecule that encodes a conserved number of genes: 13 membrane proteins from four out of five oxidative phosphorylation complexes (*cox1*-*cox3*, *cytb*, *nd1*-*nd6*, *nd4l*, *atp6* and *atp8*), two ribosomal RNA (12S and 16S) and twenty-two tRNAs^1^. However, the increasing availability of next-generation sequencing methods has revealed that the above-mentioned mtDNA description was a considerable oversimplification. Mitochondrial genomes may vary widely in size, structure, gene content, and gene order, including cases of deletion and duplication. Mitogenome sizes can be as small as 10k bp in the ctenophore *Mnemiopsis leidyi*, they can be scattered across many smaller molecules, like in the case of lice, or reach extreme levels close to 100k bp in the parasitic cnidarian *Polypodium hydriforme* due to repetitive sequences in noncoding regions^2–4^. Also, the gene content can vary greatly. There are animal mtDNA containing additional genes: *mutS* in Octocorallia^5^, *polB* in some Medusozoa and Placozoa^6,7^, *tatC* in the sponge family Oscarellidae^8^, and duplications of *atp9* or tRNA in many other species of sponges^9^. The reverse cases where some of the canonical genes are missing, are also not that uncommon. There are reports where one or two mitochondrial genes (*atp6*, *atp8*, *nd5*, tRNA) are missing^9–16^ as well as extreme cases, where all seven (*nd1-nd6* and *nd4l*) complex 1 genes have been lost^17^. Nevertheless some missing genes, such as in the case of *atp8* in bivalves^18^, could be artefacts caused by the lack of homology of particular genes to already described versions of genes in genetic databases.

Kinorhyncha, commonly known as ‘mud dragons’ are relatively under-studied groups of microscopic marine invertebrates^19,20^. These small organisms with retractable, spine-covered heads and eleven-segmented trunk are exclusively marine, comprising around 360 currently accepted species^21^, that inhabit most marine sediments, from the shallow waters to the abyssal depths. Their minute size (less than 1 mm) and subtle interspecific differences make morphological identification challenging and time-consuming. Consequently, many regions of the world, including the Arctic, likely host a substantial undescribed diversity. In the Arctic, only two kinorhynch families have been recorded so far: Echinoderidae and Pycnophyidae^22^. The family Pycnophyidae is particularly relevant to Arctic microinvertebrate biodiversity, as it is the second most diverse kinorhynch family^23^. Despite the importance of these small animals and morphological advances in classifying this phylum, molecular research on Kinorhyncha is still in its early stages^24^, and complete mitogenome data for this taxon is exceptionally limited. Up to this date, only three complete mitogenomes have been published: *Echinoderes remanei* Blake, 1930 (from synonym *Echinoderes svetlanae* Adrianov, 1999), *Setaphyes kielensis* Zelinka, 1928 (from synonym *Pycnophyes kielensis*; Sánchez, Yamasaki, Pardos, Sørensen & Martínez, 2016) and *Semnoderes armiger* Zelinka, 1928^25^. This makes Kinorhyncha one of the least represented metazoan phyla in terms of mitogenomic information. To at least partially fill this gap in the genetic information for the phylum, this study presents the sequencing and annotation of two new complete mitogenomes from the family Pycnophyidae: *Pycnophyes greenlandicus* Higgins & Kristensen, 1988 (junior synonym *Krakenella greenlandica*; for details see^26^ and *Cristaphyes cryopygus* (Higgins & Kristensen, 1988). Additionally, this study reconstructs phylogenetic relationships of twenty-two Kinorhyncha species, based on almost complete sets of mitochondrial protein coding genes, and attempt to compare differences in mitochondrial gene order among species based on complete mtDNA genomes and polycistronic transcripts.

## Results

**Fig. 1:**
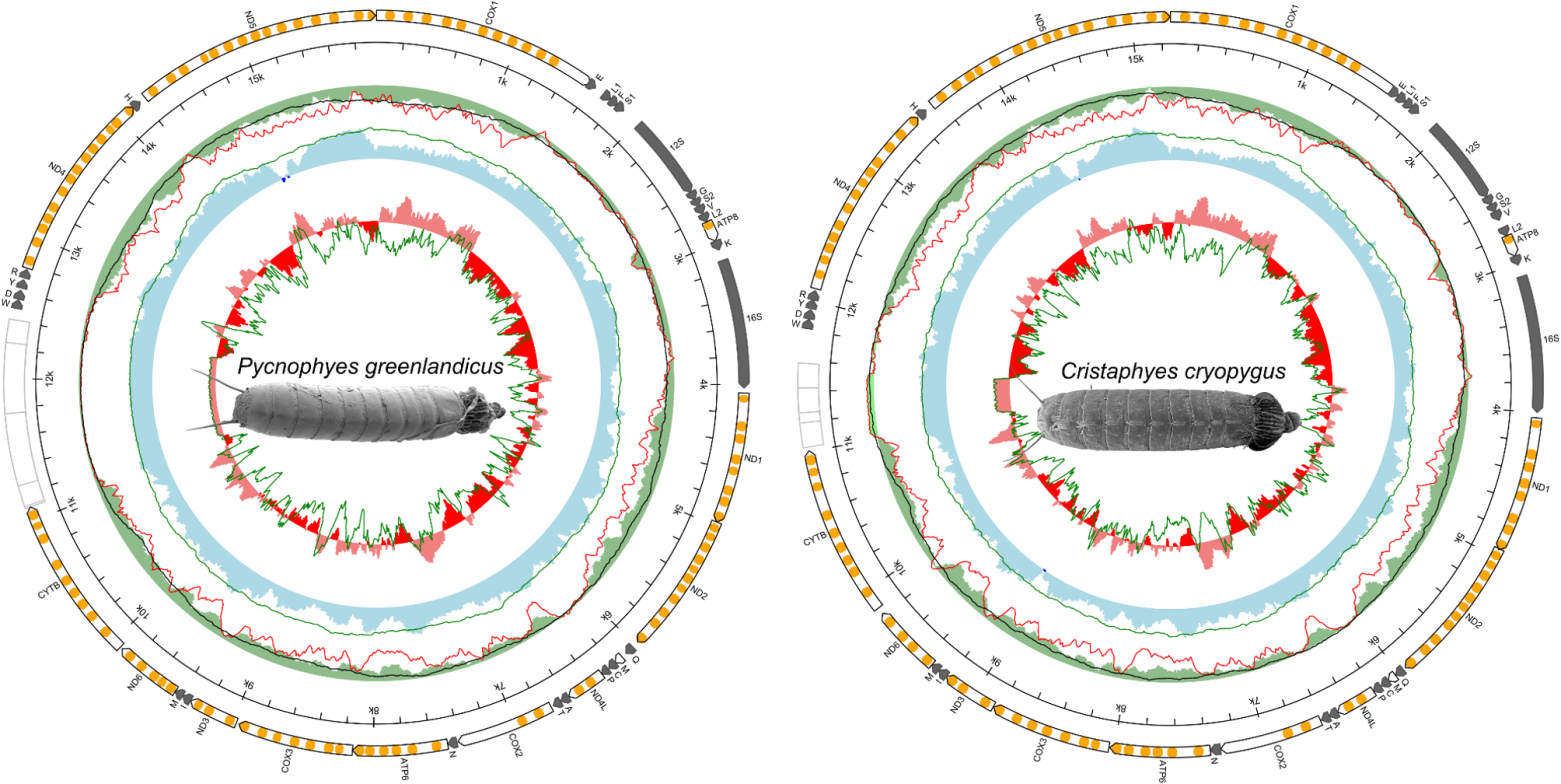
Genetic map of *Pycnophyes greenlandicus* and *Cristaphyes cryopygus* mitogenomes. The presented features follow the convention of mitoconstricor^27^. Orange ovals on arrows represent identified transmembrane domains. Grey arrows represent rRNA and tRNA. White short arrows represent duplicated tRNA-Met. Transparent light-grey boxes represent repetitive sequences in non-coding regions. Sequence local compositional bias is represented by inner circles, calculated in a 200 bp long sliding window with 25 bp steps. The first inner green circle represents local AT-skew; blue circle represents local GC-skew; red circle represents local GC content relative to average of the whole mitogenome. The red line represents filtered AT-skew at non-coding regions, and second codon position. Black and two green lines represent filtered AT-skew, GC-skew and GC content respectively, at neutral sites, calculated in a 1.000 bp long sliding window. Individuals presented in the figure show representative individuals of given species. Figures showing actual individuals deposited in Natural History Museum of Denmark can be found in supplementary data Fig. S1 and Fig S2. Full page versions of genetic maps can be found in supplementary data Fig. S4-S5.

**Fig. 2:**
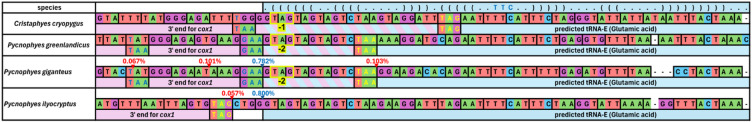
Analysis of possible *cox1* stop codon locations in four Pycnophyidae species (Two species reported in this study and two with transcriptomic data, genetically closest to *C. cryopygus* and *P. greenlandicus*). The light-pink colour represents 3’end of cytochrome c oxidase subunit I; light-blue colour represents conserved nucleotide sequence of tRNA-E; light-pink and light-blue stripes represent potential overlapping sequences; yellow brackets represent putative STOP codons in the case if mitoribosome frameshift hypothesis is true. Predicted tRNA structure and its anticodon has been shown in dot-bracket notation. In *C. cryopygus*, *P. greenlandicus* (DNA-seq) and *P. giganteus* (RNA-seq) *cox1* overlaps with tRNA-E. Numbers and arrows show transcripts with polyadenylation starting after the indicated nucleotide. Other mitochondrial genes coverage and percent of polyadenylated transcripts (*P. giganteus* and *P.* *ilyocryptus*) as well as protein alignment of COX1 kinorhynch proteins can be found in Supplementary data Table S1 and S2, and Fig. S6.

The assembled mitogenomes consist of a non-canonical number of genes: 13 protein coding genes, 2 rRNAs and 23 tRNAs (duplication of tRNA-Met) with all genes localised on the same strand (Fig. 1). The assembled mitogenome sequence length for *Cristaphyes cryopygus* was 15,259 bp and 15,958 bp for *Pycnophyes greenlandicus*. However, the true length of the sequence may differ due to repetitive sequences present in the non-coding region located between the *cytb* and tRNA-Trp. The average coverage of the sequencing reads was 206x for *P. greenlandicus* and 159x for *C. cryopygus*. Detailed read mapping plots can be found in the supplementary file Fig. S3. In both mitogenomes the *atp8* is one of the few genes with a truncated stop codon caused by excision of the tRNA-Lys and polyadenylation of the 3’ end of the gene. However, this feature seems not to be present in any of the three other available Kinorhyncha mtDNAs in which a proper stop codon exists for the *atp8* gene. In *C. cryopygus* truncated stop codons are also present in *cox1*, *cox2*, *nd3*, and *nd4l*. Interestingly, in the mitogenome of *P. greenlandicus*, the *cox1* gene seems to lack the proper stop codon (Fig. 2). The first TAA stop codon is located inside the next tRNA gene, and the first truncated (T--) stop codon option is located six codons upstream the tRNA-E gene boundaries with weak or no identifiable polyadenylation signal (data from closely related *P. giganteus* transcriptome).

The annotation procedure detected a second version of tRNA-Met. This duplicated tRNA is also present in two previously described mitogenomes *Echinoderes remanei* (*svetlanae*) and *Setaphyes* (*Pycnophyes*) *kielensis*^25^ but was not identified in *Semnoderes armiger.* The latter mtDNA sequence should be interpreted with caution due to the incomplete open reading frame for *nd5* and lack of an associated scientific article detailing the analytical procedure, attached to the GenBank record. This hints at possible deletions and assembly artefacts in this sequence. Similar duplications of mitochondrial tRNA-Met were also observed in some bivalves, amphibians, tunicates and sponges^9,28–32^. The AT content of both Kinorhyncha species is very high and exceeds 70% (73.04% *C. cryopygus* and 71.95% *P. greenlandicus*), which seems to be consistent for this phylum (76.18% *S. armiger*, 74.03% *S. kielensis*, 74.43% *E. remanei*).

**Fig. 3:**
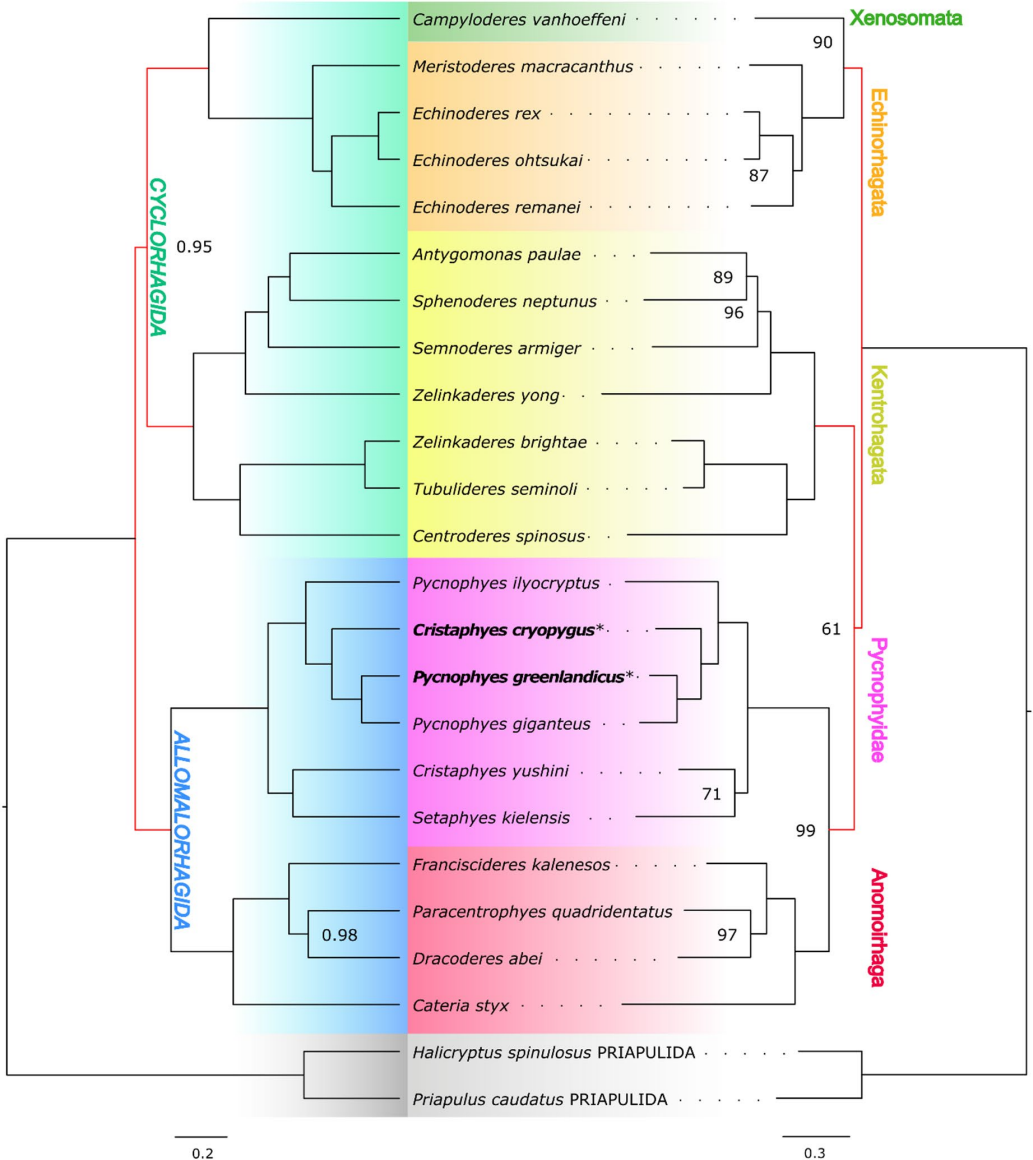
Kinorhynch phylogeny based on mitochondrial genomic data. Analysis based on eleven protein coding genes (*atp8* and *nd4l* not included). Bayesian inference posterior probabilities (left) and Maximum-likelihood bootstrap values (right) were 1 or 100 respectively, except for the nodes indicated by the actual number. Asterisks show the phylogenetic position of *Pycnophyes greenlandicus* and *Cristaphyes cryopygus*. GenBank and SRA database accession numbers for data used in phylogenetic reconstruction: SRR14509479-SRR14509496^33^, MF953591, KU975552, KU975551^25^ PX569133, PX569134 (this study).

Phylogenetic reconstruction separated kinorhynch mitochondrial sequences into five clades (*incertae sedis* order for Pycnophyidae), correctly placing *P. greenlandicus* and *C. cryopygus* into the family Pycnophyidae (Fig. 3). The changing topology close to the root of the trees between Maximum-likelihood and Bayesian inference showed limitations of mitochondrial markers in resolving deeper relations between species. The ML method, with very low bootstrap support (61 per 100 resolved trees) incorrectly reconstructed relations between mitogenomic orders clustering Kentrorhagata under Allomalorhagida class^33^. In both phylogenetic reconstructions the newly sequenced mitogenomes clustered between two other *Pycnophyes* sequences, depicting the genus *Pycnophyes* as paraphyletic and *Cristaphyes* as polyphyletic.

**Fig. 4:**
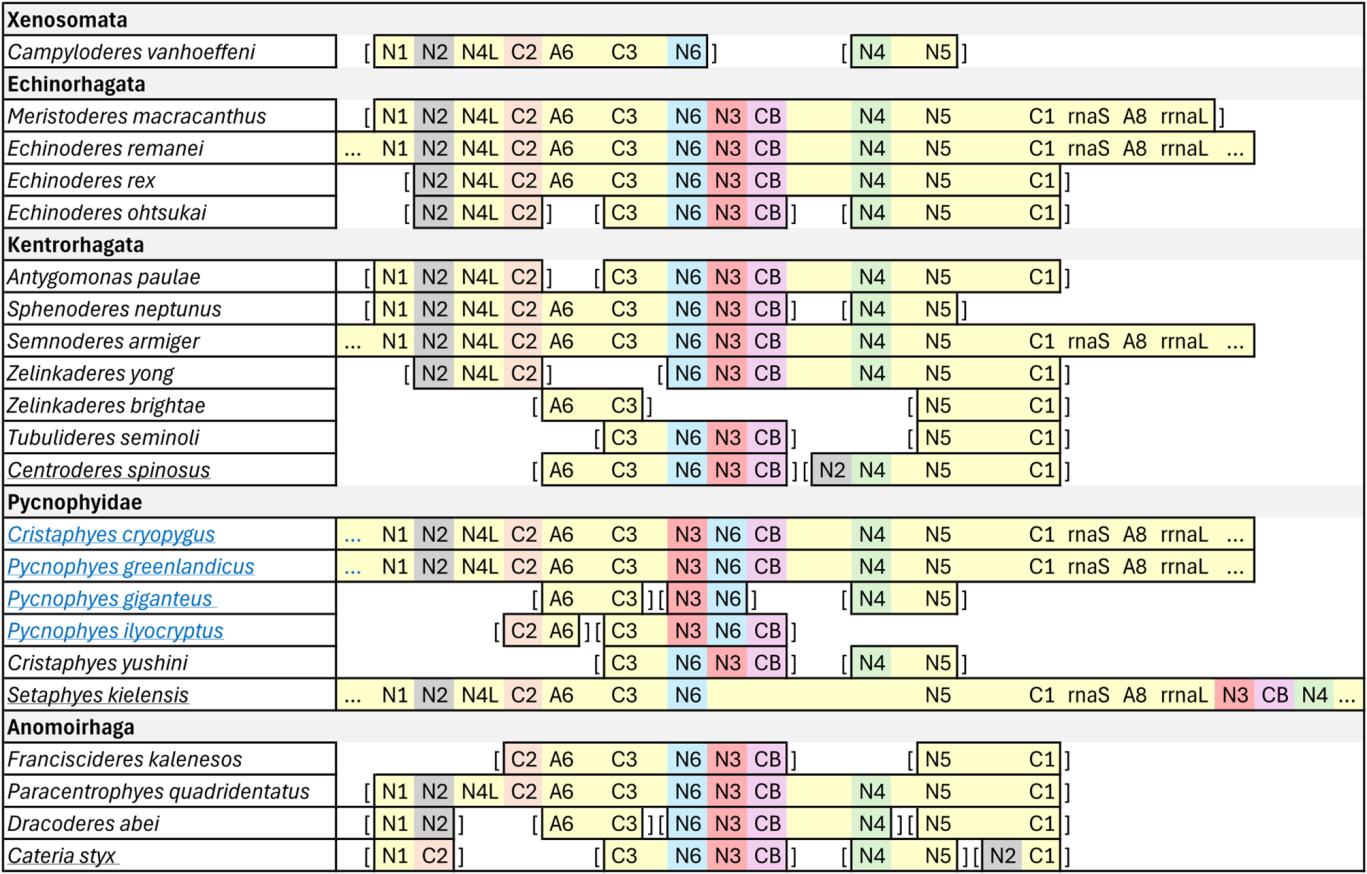
Gene order in Kinorhyncha mitogenomes and polycistronic contigs containing more than one protein coding gene, extracted from assembled transcriptomes. Square brackets show boundaries of the contigs. Different colours (other than yellow) indicate that even though available data is partial and provisionally inferred, rearrangement for a particular gene was observed in at least one of available datasets. Underlined species name indicates rearrangement in mitogenome of this species compared to assumed most common gene order (note: although gene blocks align between species, this concordance does not necessarily confirm that the order of those blocks is accurate). The blue colour in the species name indicates species with *nd6-nd3* rearrangement. Order names are kept according to previous work^33^. Gene representations have been stretched (yellow empty spaces) to better visualise similarities or lack of continuity in gene order.

The gene order of the two newly sequenced mtDNAs is identical (Fig. 4). This gene order differs from the most commonly observed in Kinorhyncha (based on very limited and incomplete available data, also excluding all tRNAs) by one main rearrangement. Neighbouring genes *nd3* and *nd6* switched places, which appears to be consistent with other two *Pycnophyes* species. However, this arrangement is not consistent with the gene order in polycistronic transcripts from *Cristaphyes yushini*. The other three species in which gene order rearrangements were observed were *Setaphyes kielensis*^34^ (previously assigned as *Pycnophyes kielensis*^25^), *Cateria styx* and *Centroderes spinosus* (Fig. 3).

## Discussion

The assembled mitogenomes showed coverage spikes in the repetitive non-coding region, making the technical assembly of this region difficult (Fig. 1). Read mapping showed twenty-one estimated repetitive 163 bp long fragments in *Pycnophyes greenlandicus* and three complete and one partial repeat unit of 163 bp length in the *Cristaphyes cryopygus* mitogenome. The true size of such mitogenomes has become a topic of growing interest in recent studies. Most commonly used systems like Illumina or Aviti provide high coverage, good quality data. However, the length of the sequencing reads makes the assembly of non-coding repetitive sequences problematic, in many cases hiding the true size of mitogenomes. Recent usage of long read sequencing technologies (PacBio, Nanopore) opened a new branch of research concerning evolution of mitogenomes^3,35–37^. Unfortunately, this approach is for now not easily achievable for single microscopic animals due to the small amount and poor quality (short fragments) of extracted DNA.

Acquired phylogenetic trees based on mitochondrial sequences are consistent up to the order level (Fig. 3). In the Maximum-likelihood method, nodes close to the root lose reliable bootstrap support making mtDNA markers less suitable in analysing relations between orders. This observation is well supported by a plethora of previous studies^36,38–40^. Animal mitochondrial genes evolve faster than nuclear ones, and with time, the accumulation of mutations starts to blur the phylogenetic signals (codons can mutate back and forth many times). Additionally, events like hybridisation, speciation followed by incomplete lineage sorting, as well as mitochondrial or nuclear introgression can cause discordance between nucDNA and mtDNA based phylogenetic trees^41–43^. However, in case of available Kinorhyncha data, if we compare phylogenetic trees based on mitochondrial sequences and trees made from transcriptomes consisting mostly of nuclear protein genes^33^we can see that species relations are almost identical (especially at the order level). Branches that differ are usually correlating with lower support (Posterior probability or Bootstrap) at nodes closer to the root (ML). The most intriguing difference between nuclear and mtDNA trees is the position of the order Xenosomata. In the nuclear tree of Herranz et al^33^. this order is sister clade to Echinorhagata + Kentrorhagata, but in trees based on mtDNA (BI), it is sister clade to only Echinorhagata, and together Echinorhagata and Xenosomata represent the sister clade to Kentrorhagata. The seemingly unstable position of Xenosomata has also been emphasised in previous papers, and for instance^44^ demonstrated that the position of the clade appeared to be highly sensitive to choice of molecular marker and analytical approach. Thus, the discrepancy between the nuclear and mtDNA trees does not come as a surprise. An improved taxon sampling from Xenosomata, including representatives of *Ryuguderes*, could perhaps solve the issue and establish the position of Xenosomata. But more importantly, despite the continued uncertainty regarding the exact position of Xenosomata, it is worth noting that the mtDNA analysis agrees with all previous molecular analyses^33^ in supporting Xenosomata as a separate evolutionary lineage, rather than a kentrorhagid ingroup as suggested in older classifications. Noteworthy, in order Pycnophyidae, the newly described mitogenome sequence for *Cristaphyes cryopygus* was placed between mitogenome sequences from the *Pycnophyes* genus, making *Cristaphyes* polyphyletic (Fig. 3). Additionally, to the phylogenetic analysis based on mitochondrial markers, the gene order, e.g. rearrangement of *nd3* and *nd6* genes, seems to support the close relationship of mitogenomes between *Cristaphyes cryopygus* and *Pycnophyes* species (Fig. 4). Such inconsistencies within and between genera indicate shortcomings in available data, regarding the taxa sampled so far. Therefore, solutions to these problems will require separate joint studies containing detailed morphological and genetic based analysis e.g. additional sampling and sequencing of closely related species.

 The most unusual feature observed in both assembled mitogenomes is the overlap of the *cox1* open reading frame (ORF) and tRNA-Glu (conserved between closely related species) located at the 3’end of the gene (Fig. 1). Most commonly, tRNAs are excised during maturation of the transcripts and later any remaining broken protein gene ORFs (without proper stop codon) are completed through polyadenylation of truncated transcripts, restoring the TAA stop codon. In the case of *C. cryopygus*, the first thymine that can complete the stop codon through polyadenylation is located only three nucleotides upstream of the predicted 5’end of tRNA-Glu, and with the absence of additional RNAseq data for this species, we are leaning towards accepting this small discrepancy. However, in case of *P. greenlandicus*, the first upstream putative stop codon is located 17 nucleotides from the 5’end of the tRNA. A parallel situation was detected in closely related transcriptomic data from *P. giganteus* but not in the *cox1* gene from the *P. ilyocryptus* transcriptome (Fig. 2). With the help of RNAseq data, we have mapped polyadenylated reads and identified the highest poly(A) signal level exactly at the 5’end of predicted tRNA-Glu, indicating GAA as a potential stop codon in this species or just ‘protection signal’ from transcript degradation. The poly(A) signal level in *P. giganteus* was similar to the polyadenylation of *cox1* in *P. ilyocryptus* but in comparison with other mtDNA genes, the detected level was not very convincing (Supplementary data Table. S1 and S2). In humans, alternative stop codons AGA/AGG were thought to be used for *cox1* and *nd6* genes^45,46^. Based on that, we checked if similarly to human mtDNA, the GAA codon or any other codon around the 3’end of the *cox1* gene is not used in protein translation (codon usage equalled zero) (Supplementary data Table S3 and S4). Unfortunately, this was not the case. However, the study by Temperley el al. showed that in those two human mitochondrial genes a mitoribosomal frameshifts might occur forcing utilisation of out of frame stop codons^47^. Authors speculated that among others those frameshifts can be promoted by upstream “slippery” sequences or downstream stable secondary structures like tRNAs. Another possibility is that, we may have detected a phenomenon somewhat analogous to that observed in Octocorallia, which lacks conventional stop codons^48^. Alternatively, specialised rescue protein systems may facilitate termination and dissociation of the translation complex stuck at the end of the transcript^45,49^, or only a subset of transcripts may be translated while the remainder are processed to release the tRNA-Glu gene. Resolving this uncertainty will require further investigation, particularly through proteomic analyses.

## Conclusion

The taxonomy of Kinorhyncha constantly undergoes reclassification^34^, and the newly acquired data, as well as two new mitogenomes greatly contribute to the cause. Mitogenome sequencing and consecutive analyses allowed us to further investigate the diversity of Kinorhyncha from a broader molecular perspective. Before, the phylogenetic approach was focused on morphological features governing the taxonomy, systematics and classification. However, recent technological developments facilitated the process of sampling, laboratory procedures and acquisition of data with high resolution. Our results report a few interesting features present in the mtDNA molecules of *P. greenlandicus* and *C. cryopygus*, such as unusual overlap between the *cox1* open reading frame (ORF) and tRNA-Glu gene, and rearrangement of *nd3* and *nd6* genes. Moreover, with the steady inflow of new data the phylogeny of the orders seems to be getting resolved better and better, with an exception of Xenosomata, for which the molecular markers and methods used so far presented contradictory positions^33,44^. The polyphyletic origin of *Cristaphyes* genus displayed on Fig. 3 is another example of plausible misclassification. Both phylogenetic entanglements are most likely caused by undersampling of taxa in situ and the total diversity of the phylum remaining unknown and underestimated. To further improve the classification, investigations such as the present one, utilising high resolution molecular data, are necessary to understand the phylogenetic relationships within and among mud dragon communities. Implementing genetic, or soon even genomic data is mandatory both in new species descriptions and existing taxonomic revisions.

While high-throughput metabarcoding is gaining increasing attention in the scientific community, it is important to remember that results of such studies are highly dependent on the quality of applied genetic databases^50–52^. These databases would not exist and could not be expanded without the hard work of taxonomists and molecular biologists focused on the reliable description of individual species. One such source of conserved sequences used in metabarcoding and phylogenetic studies (e.g. cytochrome c oxidase subunit I gene) are complete mitochondrial genomes.

## Methods

Samples containing examined species were collected with Van Veen grab during two Arctic expeditions to Disko Island (Western Greenland) in 2023 and Western Spitsbergen of Svalbard archipelago in 2024. Detailed sampling information can be found in Table 1. Individual specimens were extracted from the sediments using the bubble and blot method^53^, sorted under a stereomicroscope, and preserved in 96% ethanol at −20 °C until further analysis. Upon the DNA extraction procedure using the Tissue DNA Purification Kit (EURx) with an additional step of exoskeleton retention from the lysis buffer^54^, individual specimens were classified to species level using a light microscope.

**Table 1:** Sampling information for species used in this study.

Name	Date	Area	Position	Depth [m]
*C. cryopygus*	August 2023	North Fjord, Disko Island, Greenland	69°56.524′N 54°24.736′W	103
*P. greenlandicus*	August 2024	Kongsfjord, Spitsbergen, Svalbard	78°58.680′N 11°42.360′E	300

Two specimens from two species (a male *Pycnophyes greenlandicus* and a male *Cristaphyes cryopygus*) were chosen for further molecular analysis. After DNA extraction, the recovered cuticles of the two specimens were deposited as hologenophores at the Natural History Museum of Denmark (NHMD). *Pycnophyes greenlandicus* hologenophore was mounted for light microscopy in Fluoromount-G, between two cover glasses attached to an H-S plastic slide and stored under museum catalogue number NHMD-1986914. *Cristaphyes cryopygus* hologenophore was critical point dried, mounted on an aluminium stub, sputter coated with gold and examined with a Zeiss Sigma 360VP scanning electron microscope (SEM). This hologenophore is stored under catalogue number NHMD-1986932 (Supplementary Fig. S1 and S2). It is worth noting that those two species were recently redescribed and synonymised (*C. scatha* as a junior synonym of *C. cryopygus*, and *P. ancalagon* as a junior synonym of *P.greenlandicus*), therefore the scientific names used in this article are in accordance with Sørensen et al^26^..

DNA isolated from single individuals was sent for next-generation total DNA sequencing to GENOMED (Poland), where the DNA concentrations were measured on an Infinite apparatus (Tecan): 1.05 ng/µl for *P. greenlandicus* and 0.56 ng/µl for *C. cryopygus*. The sequencing library was made with the NEBNext^®^ Ultra™ II DNA Library Prep Kit for Illumina^®^ (New England Biolabs) and the NGS sequencing of 2 × 150 bp paired-end was run on Aviti (Element Biosciences). Raw sequencing reads, 95 M for *C. cryopygus* and 151 M for *P. greenlandicus* were trimmed in TRIMMOMATIC^55^ and assembled with NOVOPlasty^56^ using PX205375 and PV802827 partial *cox1* sequences as a seed^26^. The assembled contigs were first verified by raw reads mapping, then annotated and visualised with MITOS2^57^ and MITOCONSTRICTOR scripts^27^ using invertebrate mitochondrial code (translation table 5). The above mentioned tools use, among others, BLAST^58^ and Wise2^59^ software for protein prediction, Infernal^60^ for rRNA prediction and ARWEN^61^ for tRNA prediction. Newly assembled sequences have been deposited in GenBank under PX569133 and PX569134 accession numbers.

Due to the limited availability of Kinorhyncha mitogenomic sequences (^25^ and MF953591), mitochondrial sequences derived from previously published transcriptomic datasets have been utilized in phylogenetic analyses^33^. Seventeen transcriptomic datasets have been downloaded from the SRA database (accession numbers can be found in the caption for Fig. 3), trimmed with TRIMMOMATIC^55^ and assembled in the CLC genomic workbench. The assembled contigs were then searched for mitochondrial genes with the help of the Wise2 tool^59^. HMM profiles were extracted from the MITOCONSTRICTOR annotation tool and later remade in HMMER^62^, based on identified sequences, to better match mitochondrial kinorhynch proteins. In cases where there was more than one contig per mitochondrial gene, sequences were discriminated with the BLAST tool^63^, simple neighbour-joining phylogenetic reconstruction in MEGA^64^, and by sequencing reads mapping (contigs with highest coverage were assumed to be the correct ones). The remaining missing or incomplete genes were assembled with NOVOPlasty^56^. Missing seed sequences were identified by lenient mapping (length fraction and similarity fraction parameters set to 0.6) of sequencing reads on already extracted mitochondrial gene alignments. Alignments of eleven mitochondrial protein-coding genes were deposited at 10.6084/m9.figshare.29269373 for future use. At the design step, rRNA genes were excluded due to the difficulties in identification of proper gene boundaries and potential insertions caused by contaminations and sequencing artefacts. The *atp8* and *nd4l* genes were excluded due to their short length, which made them difficult to reliably identify in all data sets. In a few cases the 5’ and 3’ends of the genes may be incomplete. Lastly, the incomplete *nd5* gene in the *Semnoderes armiger* mitogenome MF953591 has been recovered from the SRR14509494 transcriptomic data. Identification of the polyadenylation signal in mitochondrial contigs of *P. giganteus* SRR14509496 and *P. ilyocryptus* SRR14509495 has been done by reads mapping on *cox1* transcripts in the CLC Genomic Workbench. Detailed per-base coverage information was extracted in tab delimited format and analysed by counting the number of consecutive adenines after the possible canonical and truncated STOP codons in the 3’end region of the *cox1* gene. Next numerical information was visually verified by the presence of polyadenylated transcripts in the reads mappings (CLC Genomic workbench).

Phylogenetic reconstructions were performed in parallel Maximum-likelihood (ML) in iqTree2^65^ and Bayesian inference (BI) in Beast2^66^. Mitochondrial gene alignments were conducted in MEGA7 software^64^ with ClustalW codons alignment algorithm^67^ and all Gap Opening Penalties set to 6. Appropriate phylogenetic models were chosen based on the bModelTest tool^68^ for Beast2 and ModelFinder for iqTree2. For the phylogenetic analysis in Beast2, the TN93 + I +4G substitution model was used for all 11 gene partitions, with relaxed log-normal clock and Yule prior to the common tree. The Markov Chain Monte Carlo (MCMC) was run in 4 separate replicates for 10 million generations sampled every 10,000th step. The convergence of results between runs with 10% trees burnin was checked in Tracer^69^. The effective sample size for the estimated parameters exceeded 200. For the IqTree2, substitution model was GTR + F +I +4G for *atp6*, *cox3*, *nd1*, *nd5*; GTR + F +4R for *cytb*, *nd2*, *nd4* and *nd5*; TN + F +I +4G for *nd3* and TIM + F +4R for *cox1* and *cox2*. Phylogenetic reconstruction was run under default parameters with the ultra-fast bootstrap approximation set to 10,000 repeats.

## Supplementary Information

Below is the link to the electronic supplementary material.

Supplementary Material 1

## Data Availability

Mitogenomic sequences reported in this study, as well as raw NGS data, can be found in GenBank under PX569133, PX569134, SRR36015645 and SRR36015646 accession numbers. Alignments for mitochondrial genes extracted from transcriptomic data are available on FigShare dx.doi.org/10.6084/m9.figshare.29269373. Metadata for individuals sequenced in this study can be found in GeoNetwork database under https://doi.org/10.48457/IOPAN.2025.532.
