# Supplementary material for: Towards a mitogenomic phylogeny of mud dragons (Kinorhyncha): two new mitogenomes from the Pycnophyidae family
Source: Sci Rep. 2026 May 23;16:23684. doi: 10.1038/s41598-026-54168-x (PMC13424098; doi:10.1038/s41598-026-54168-x)
Supplement: Supplementary file 1 — Supplementary Material 1 [file 41598_2026_54168_MOESM1_ESM.pdf]

Supplementary data for:

Towards a mitogenomic phylogeny of mud dragons (Kinorhyncha): two new mitogenomes from the Pycnophyidae family.

Authors: Lubośny M., Zalewska A., Herranz M., Sørensen MV., Grzelak K.

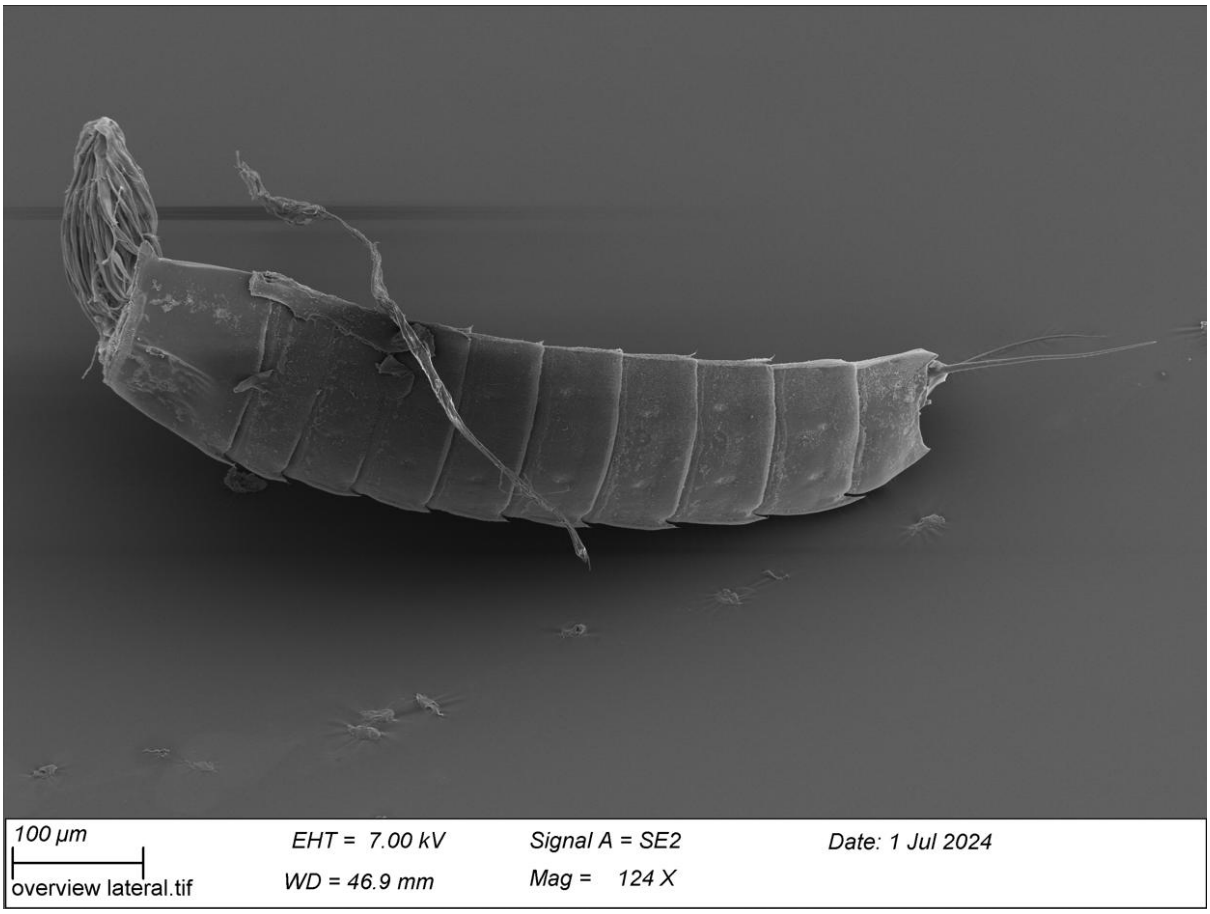

Fig S1. Scanning electron microscopy (SEM) image of *Cristaphyes cryopygus* individual used in this study.

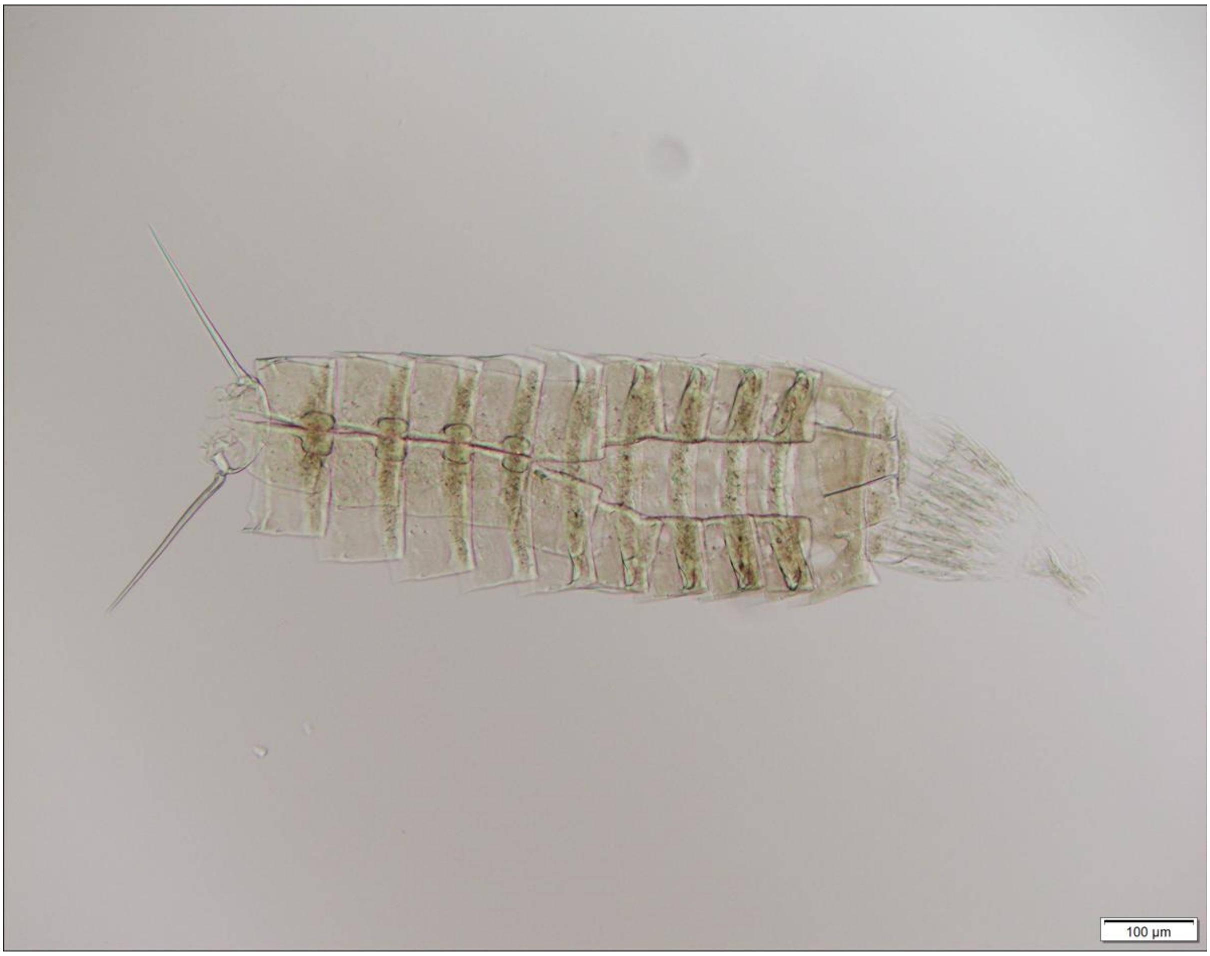

Fig S2**.** Light microscopy image of *Pycnophyes greenlandicus* individual used in this study.

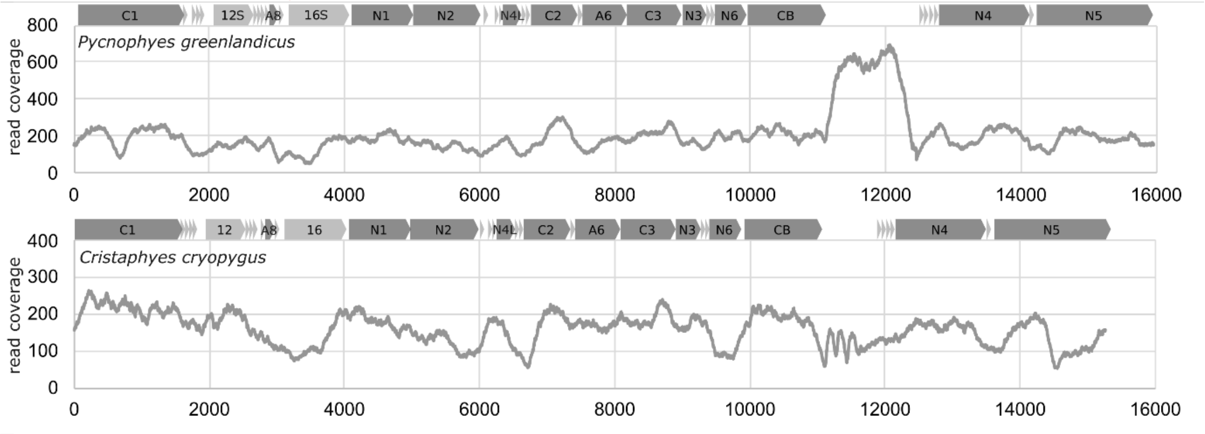

Fig S3. Sequencing coverage plot for *P. greenlandicus* and *C. cryopygus* mitogenomes.

Table S1. Gene coverage and polyadenylation signal coverage for *P. ilyocryptus* (SRR14509495) mitochondrial transcripts. Polyadenylation counted as the average number of adenine nucleotides in up to 20 bp window, excluding coverage spikes caused by adenines in non-polyadenylated reads in polycistronic transcripts.

P. ilyocryptus	Poly(A) coverage	Gene coverage	Poly(A)/Gene
cox1 TAG (STOP)	50.38	89108.26	0.057%
cox1 4bp (downstream)	712.62	89108.26	0.800%
cox2 TAG (STOP)	6.50	14665.62	0.044%
cox2 6bp (downstream)	1001.85	14665.62	6.831%
cox3 TAG (STOP)	16.55	107787.63	0.015%
cox3 23bp (downstream)	2757.35	107787.63	2.558%
atp6 TAA (STOP)	935.40	5891.97	15.876%
cytb TAG (STOP)	3.20	4170.09	0.077%
nad1 TAA (STOP)	1171.71	4686.7	25.001%
nad2 (3'end assembly problem)	n.d.	789.79	n.d.
nad3 TAA (STOP)	1785.52	14588.03	12.240%
nad4 TAA (STOP)	823.90	22387.22	3.680%
nad5 TAA (STOP)	31.95	1031.31	3.098%
nad6 TAG (STOP)	71.18	17862.57	0.398%
nad6 41bp (downstream)	1078.55	17862.57	6.038%

Table S2. Gene coverage and polyadenylation signal coverage for *P. giganteus* (SRR14509496) mitochondrial transcripts. Polyadenylation counted as the average number of adenine nucleotides in up to 20 bp window, excluding coverage spikes caused by adenines in non-polyadenylated reads in polycistronic transcripts.

P. giganteus	Poly(A) coverage	Gene coverage	Poly(A)/Gene
cox1 TAA (STOP) in tRNA-E	12.57	12168.17	0.103%
cox1 15bp (upstream) GAA	95.17	12168.17	0.782%
cox1 20bp (upstream) ATA	12.29	12168.17	0.101%
cox1 30 (upstream) TACT	8.2	12168.17	0.067%
cox2 TAA (STOP)	98.76	14135.74	0.699%
cox2 14bp (downstream)	1076.62	14135.74	7.616%
cox3 TAG (STOP)	11.67	19546.15	0.060%
cox3 6bp (downstream)	1437.00	19546.15	7.352%
atp6 TAA (STOP)	402.40	1704.21	23.612%
cytb TAG (STOP)	11.00	636.43	1.728%
nad1 TAA (STOP)	103.32	398.48	25.928%
nad2 TAA (STOP)	1.50	962.33	0.156%
nad2 21bp (downstream)	142.72	962.33	14.831%
nad3 TAA (STOP)	141.43	969.18	14.593%
nad4 TAG (STOP)	2.90	3984.65	0.073%
nad4 37bp (downstream)	288.57	3984.65	7.242%
nad5 TAA (STOP)	244.33	1719.22	14.212%
nad6 TAG (STOP)	0.14	1510.35	0.009%

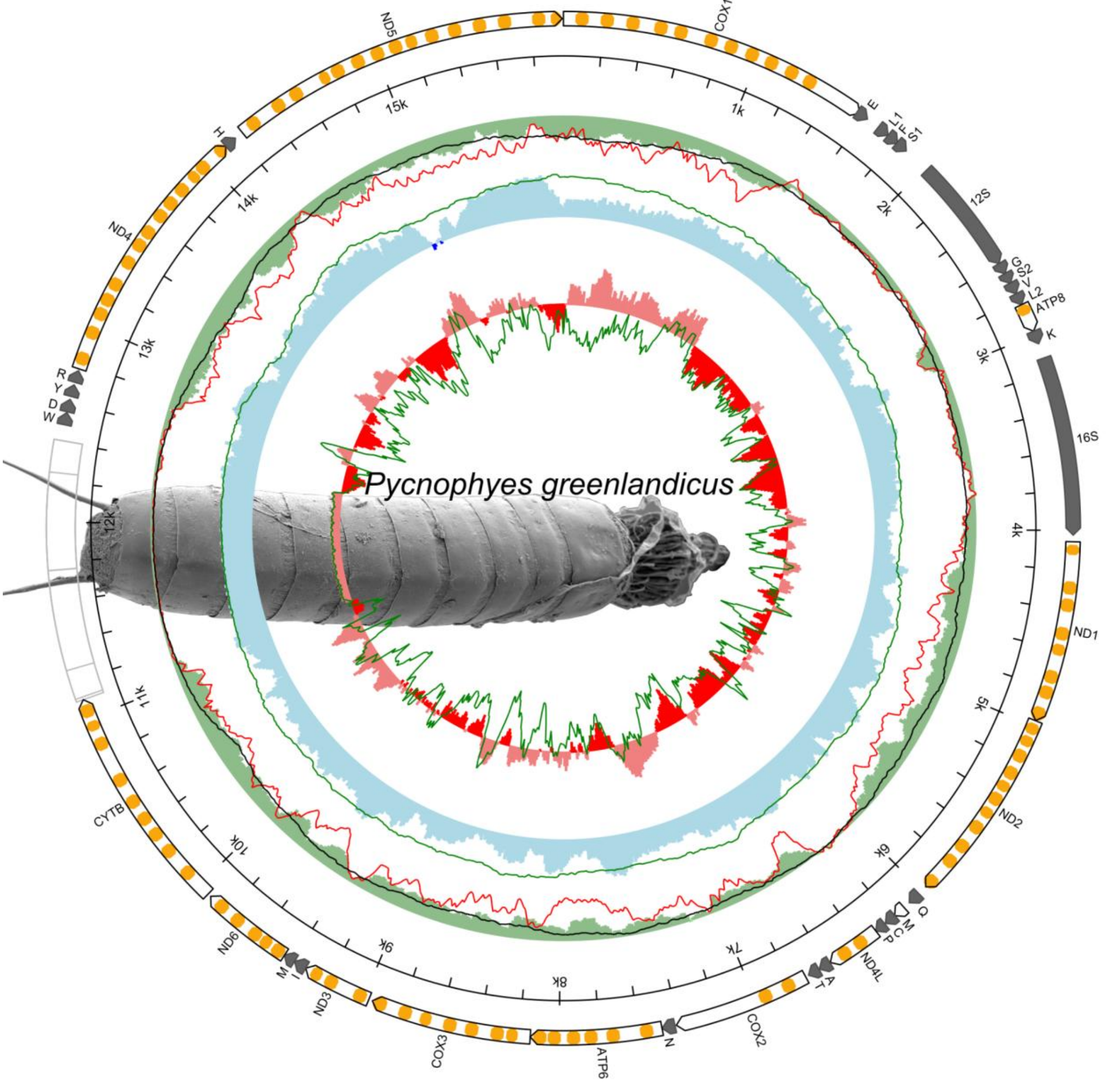

Fig S4. Genetic map of the *Pycnophyes greenlandicus* mitogenome. The presented features follow the convention of MITOCONSTRICTOR. Orange ovals on arrows represent identified transmembrane domains. Grey arrows represent rRNA and tRNA. Transparent white short arrow represent duplicated tRNA-Met. Transparent light-gray boxes represent repetitive sequences in non-coding regions. Sequence local compositional bias is represented by inner circles, calculated in a 200 bp long sliding window with 25 bp steps. The first inner green circle represents local AT-skew; blue circle represents local GC-skew; red circle represents local GC content relative to average of the whole mitogenome. The red line represents filtered AT-skew at non-coding regions, and second codon position. Black and two green lines represent filtered AT-skew, GC-skew and GC content respectively, at neutral sites, calculated in a 1.000 bp long sliding window.

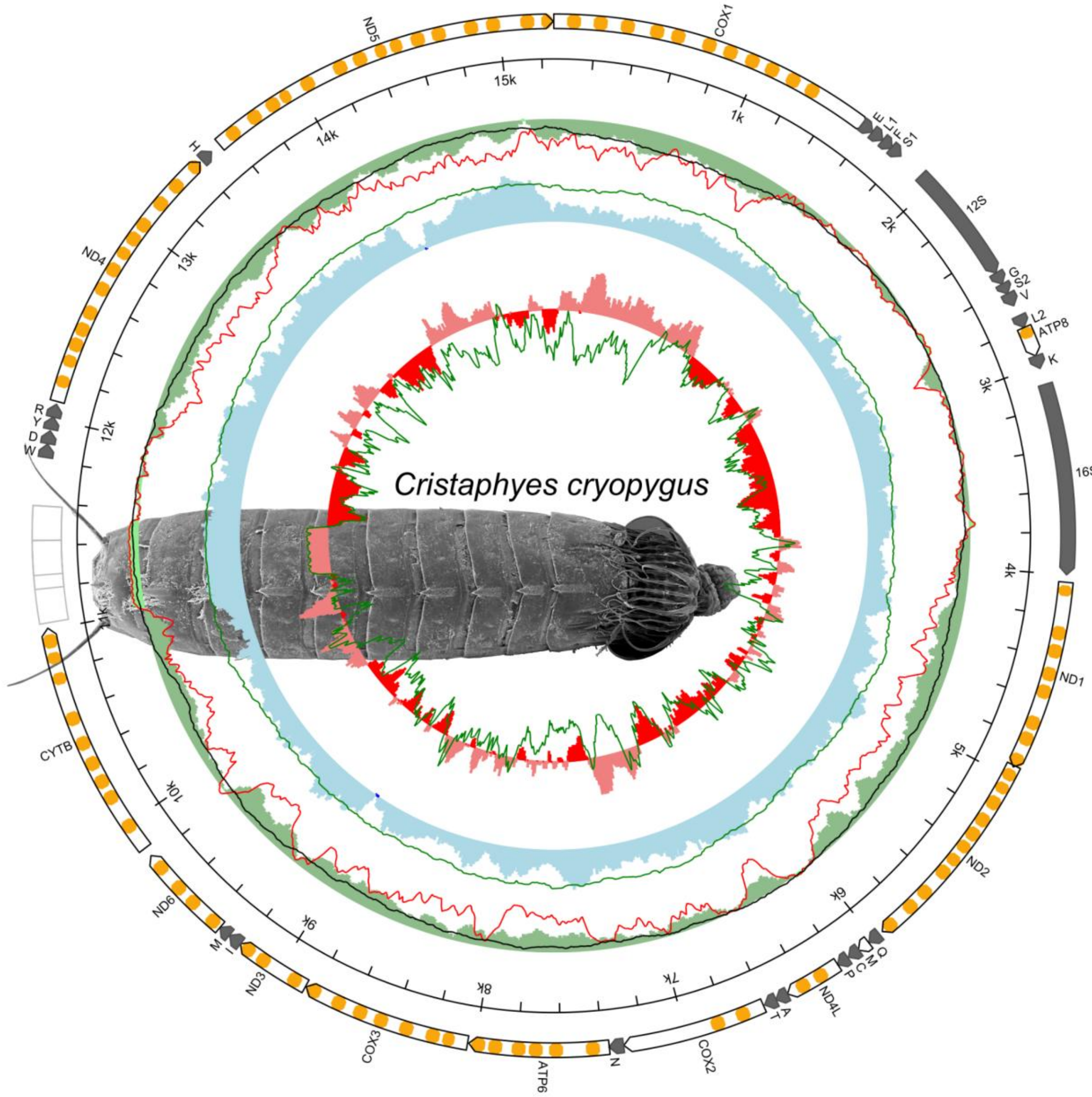

Fig S5. Genetic map of the *Cristaphyes cryopygus* mitogenome. The presented features follow the convention of MITOCONSTRICTOR. Orange ovals on arrows represent identified transmembrane domains. Grey arrows represent rRNA and tRNA. Transparent white short arrow represent duplicated tRNA-Met. Transparent light-gray boxes represent repetitive sequences in non-coding regions. Sequence local compositional bias is represented by inner circles, calculated in a 200 bp long sliding window with 25 bp steps. The first inner green circle represents local AT-skew; blue circle represents local GC-skew; red circle represents local GC content relative to average of the whole mitogenome. The red line represents filtered AT-skew at non-coding regions, and second codon position. Black and two green lines represent filtered AT-skew, GC-skew and GC content respectively, at neutral sites, calculated in a 1.000 bp long sliding window.

Table S3. The codon usage for *Cristaphyes cryopygus* mtDNA genes. Fields marked with color indicate codons that are not used in a particular gene. Codons marked with red indicate codons found at the 3’ end of *cox1* open reading frame.

	AAA (K)	AAC (N)	AAG (K)	AAT (N)	ACA (T)	ACC (T)	ACG (T)	ACT (T)	**AGA (S)**	AGC (S)	**AGG (S)**	**AGT (S)**	ATA (M)	ATC (I)	ATG (M)	**ATT (I)**
cox1	2	1	4	15	1	0	1	28	7	0	4	9	22	1	5	35
cox2	3	1	2	9	0	0	1	5	6	0	3	2	15	0	3	22
cox3	2	0	1	8	2	0	0	11	7	2	5	2	10	0	1	18
cytb	10	1	2	11	2	0	0	9	10	0	5	5	18	3	6	20
atp6	2	0	2	4	2	0	0	4	8	0	4	5	19	0	2	11
atp8	1	1	0	1	0	0	0	0	4	0	1	3	3	0	1	3
nd1	5	0	3	5	0	1	0	3	4	0	1	1	13	1	5	27
nd2	4	1	3	6	3	0	0	2	20	1	10	7	19	6	7	20
nd3	3	0	0	1	0	0	0	1	1	0	2	1	7	0	1	12
nd4	11	1	1	6	1	0	0	4	12	1	8	12	28	2	10	22
nd4l	0	0	1	1	0	0	0	1	2	0	2	1	6	1	2	10
nd5	9	2	6	14	5	1	0	8	17	1	10	8	39	1	10	34
nd6	1	1	3	0	0	0	0	1	2	0	1	3	10	1	3	8

	CAA (Q)	CAC (H)	CAG (Q)	CAT (H)	CCA (P)	CCC (P)	CCG (P)	CCT (P)	CGA (R)	CGC (R)	CGG (R)	CGT (R)	**CTA (L)**	CTC (L)	CTG (L)	CTT (L)
cox1	2	0	2	18	3	1	1	14	7	0	4	2	1	0	1	12
cox2	5	0	0	4	1	0	0	9	2	0	4	2	1	0	0	3
cox3	5	0	1	10	1	0	1	4	1	0	4	1	5	0	2	1
cytb	2	2	5	6	0	0	2	12	4	0	1	3	5	1	1	2
atp6	0	0	2	2	2	0	0	3	2	0	1	0	2	0	0	1
atp8	0	0	1	0	0	0	0	2	0	0	0	0	1	0	0	0
nd1	2	0	1	1	2	1	1	4	4	0	2	2	3	0	0	4
nd2	1	0	0	1	0	1	0	3	2	0	1	1	4	0	1	3
nd3	1	0	1	1	0	0	0	3	0	0	0	0	2	0	0	0
nd4	1	0	2	5	0	0	1	7	1	0	4	5	7	0	0	9
nd4l	0	0	1	1	0	0	0	0	1	0	1	0	0	0	0	0
nd5	3	0	5	3	0	0	0	8	3	0	3	2	2	0	2	5
nd6	0	0	0	0	0	1	0	1	1	0	0	0	3	1	0	3

	GAA (E)	GAC (D)	GAG (E)	GAT (D)	GCA (A)	GCC (A)	GCG (A)	GCT (A)	GGA (G)	GGC (G)	GGG (G)	**GGT (G)**	**GTA (V)**	GTC (V)	GTG (V)	GTT (V)
cox1	6	2	6	14	8	0	0	14	28	2	15	21	15	1	9	22
cox2	5	1	2	8	1	0	0	2	6	1	9	2	8	1	5	10
cox3	5	1	1	3	2	0	1	6	8	1	4	7	11	2	7	8
cytb	5	1	1	8	4	1	3	10	8	1	9	10	14	1	5	19
atp6	3	1	1	4	0	0	1	4	1	2	5	4	9	1	2	17
atp8	0	0	0	0	0	0	0	0	1	0	0	0	1	1	1	3
nd1	7	0	5	5	6	0	0	5	8	1	8	8	11	0	6	11
nd2	2	0	6	4	0	0	0	1	3	2	11	7	11	0	9	12
nd3	4	0	2	3	1	0	1	0	1	1	4	1	5	0	2	2
nd4	9	0	4	4	1	0	0	9	18	1	8	12	14	1	7	11
nd4l	3	1	1	1	1	0	1	1	3	0	2	1	5	0	3	3
nd5	5	1	6	17	6	1	1	10	19	0	17	15	11	0	5	23
nd6	1	0	1	2	1	0	0	2	5	0	3	7	3	0	1	10

	TAC (Y)	**TAT (Y)**	TCA (S)	TCC (S)	TCG (S)	TCT (S)	TGA (W)	TGC (C)	**TGG (W)**	TGT (C)	TTA (L)	TTC (F)	TTG (L)	**TTT (F)**
cox1	1	15	3	2	1	17	11	0	8	3	40	1	12	48
cox2	2	11	1	0	1	5	4	1	6	6	9	0	9	10
cox3	0	8	3	0	3	7	7	1	5	1	25	1	3	33
cytb	3	22	1	0	2	2	8	0	4	5	33	2	12	39
atp6	1	5	2	0	0	4	3	1	3	5	26	2	7	29
atp8	0	3	0	0	0	2	2	0	0	0	3	0	1	8
nd1	2	10	3	1	1	10	4	0	2	2	28	4	11	48
nd2	3	13	1	2	0	5	3	0	2	2	39	3	7	57
nd3	1	3	3	0	0	7	2	0	1	1	16	2	2	20
nd4	3	17	4	0	0	13	6	0	5	14	46	2	14	59
nd4l	0	2	0	1	0	1	4	0	1	2	7	0	4	12
nd5	1	22	2	1	2	20	10	0	6	5	55	1	27	81
nd6	1	7	2	1	2	3	1	0	2	2	19	0	5	31

Table S4. The codon usage for *Pycnophyes greenlandicus* mtDNA genes. Fields marked with color indicate codons that are not used in a particular gene. Codons marked with red indicate codons found at the 3' end of *cox1* open reading frame.

	AAA (K)	AAC (N)	**AAG (K)**	AAT (N)	ACA (T)	ACC (T)	ACG (T)	ACT (T)	**AGA (S)**	AGC (S)	AGG (S)	AGT (S)	ATA (M)	ATC (I)	ATG (M)	ATT (I)
cox1	2	1	5	14	4	0	2	24	10	0	4	1	20	0	7	34
cox2	3	1	1	7	0	0	1	4	8	0	2	1	8	1	7	21
cox3	4	1	0	6	1	1	0	8	12	0	1	4	8	1	2	14
cytb	6	1	6	10	0	0	1	8	10	0	8	7	18	3	4	19
atp6	3	0	1	3	0	0	0	5	7	1	8	3	18	1	3	13
atp8	0	0	1	0	0	0	0	0	2	0	3	2	1	0	0	1
nd1	8	0	1	5	0	0	1	3	4	0	3	0	13	3	5	30
nd2	4	1	7	4	0	0	1	2	12	0	7	7	20	0	7	28
nd3	2	0	0	1	1	0	0	1	3	1	0	2	9	0	1	9
nd4	5	0	3	11	1	0	0	6	10	0	11	7	25	2	7	28
nd4l	0	1	0	0	0	0	0	0	4	0	0	1	6	0	1	12
nd5	11	1	6	13	3	1	0	9	14	1	9	7	28	1	6	39
nd6	3	0	1	2	1	0	0	3	3	1	2	2	7	1	4	8

	CAA (Q)	CAC (H)	CAG (Q)	CAT (H)	CCA (P)	CCC (P)	CCG (P)	CCT (P)	CGA (R)	CGC (R)	CGG (R)	CGT (R)	CTA (L)	CTC (L)	CTG (L)	CTT (L)
cox1	3	0	1	18	1	2	0	16	4	0	3	6	2	0	2	9
cox2	3	0	2	4	1	0	0	9	3	0	2	3	2	0	0	3
cox3	3	0	2	11	0	0	0	6	1	1	3	1	1	0	0	5
cytb	4	1	2	7	2	1	2	9	2	0	2	4	0	1	0	4
atp6	0	1	3	1	1	0	0	4	2	0	1	0	3	0	1	1
atp8	1	0	0	0	0	0	0	2	0	0	0	0	0	1	0	0
nd1	2	0	1	1	1	1	0	6	3	0	4	1	1	0	0	5
nd2	1	0	0	0	0	1	0	3	3	0	1	0	3	0	2	8
nd3	2	0	0	1	0	0	0	3	0	0	0	0	0	0	0	2
nd4	3	0	0	5	1	1	0	6	1	0	3	4	4	0	0	11
nd4l	0	0	0	1	0	0	0	0	1	0	0	1	0	0	1	0
nd5	6	1	3	2	1	1	1	4	1	0	2	5	3	0	1	13
nd6	0	0	0	0	0	0	0	2	0	0	0	1	2	0	1	1

	**GAA (E)**	GAC (D)	GAG (E)	GAT (D)	GCA (A)	GCC (A)	GCG (A)	GCT (A)	GGA (G)	GGC (G)	**GGG (G)**	GGT (G)	**GTA (V)**	**GTC (V)**	**GTG (V)**	**GTT (V)**
cox1	9	2	4	14	6	1	2	14	19	4	22	20	16	1	10	26
cox2	4	0	5	8	0	0	2	1	8	0	6	7	14	0	2	10
cox3	1	0	4	6	4	0	0	5	9	1	6	4	12	0	5	16
cytb	2	1	3	10	1	2	0	12	9	0	12	11	11	2	10	18
atp6	1	1	2	5	0	0	0	4	10	0	3	1	7	1	6	15
atp8	1	0	1	1	0	0	0	0	2	0	1	0	1	0	1	2
nd1	7	1	5	4	3	0	1	5	10	1	7	6	7	1	7	11
nd2	6	0	5	5	1	0	0	2	7	0	11	8	13	3	4	13
nd3	4	0	2	3	1	0	0	0	1	0	3	3	4	1	0	6
nd4	5	0	6	7	1	1	1	7	14	4	9	18	6	3	6	26
nd4l	3	0	1	1	0	0	0	2	3	0	5	1	4	0	2	6
nd5	4	4	10	13	6	1	1	10	22	0	17	15	14	3	9	24
nd6	0	0	3	1	2	0	0	2	8	0	3	2	4	0	6	9

	TAC (Y)	**TAT (Y)**	TCA (S)	TCC (S)	TCG (S)	TCT (S)	TGA (W)	TGC (C)	TGG (W)	TGT (C)	TTA (L)	TTC (F)	TTG (L)	TTT (F)
cox1	0	16	3	2	2	15	10	1	8	2	38	1	16	45
cox2	2	9	2	2	0	4	7	1	3	6	12	0	5	12
cox3	3	4	2	1	2	8	7	1	7	2	25	3	3	31
cytb	3	20	0	0	0	7	5	0	6	4	19	5	25	46
atp6	0	7	2	0	0	3	3	0	1	5	25	2	5	26
atp8	0	3	0	0	0	1	0	0	0	1	5	0	3	11
nd1	1	11	3	1	0	9	4	0	2	5	20	3	17	50
nd2	2	13	3	0	0	9	1	0	5	1	27	3	12	56
nd3	0	5	0	1	1	4	2	0	1	2	12	2	6	14
nd4	1	20	0	0	0	15	8	1	3	11	34	2	18	65
nd4l	0	3	0	1	0	1	4	0	1	2	8	1	6	7
nd5	2	18	2	0	1	20	9	1	5	7	61	2	19	81
nd6	0	7	3	0	0	2	4	0	2	4	15	3	6	25

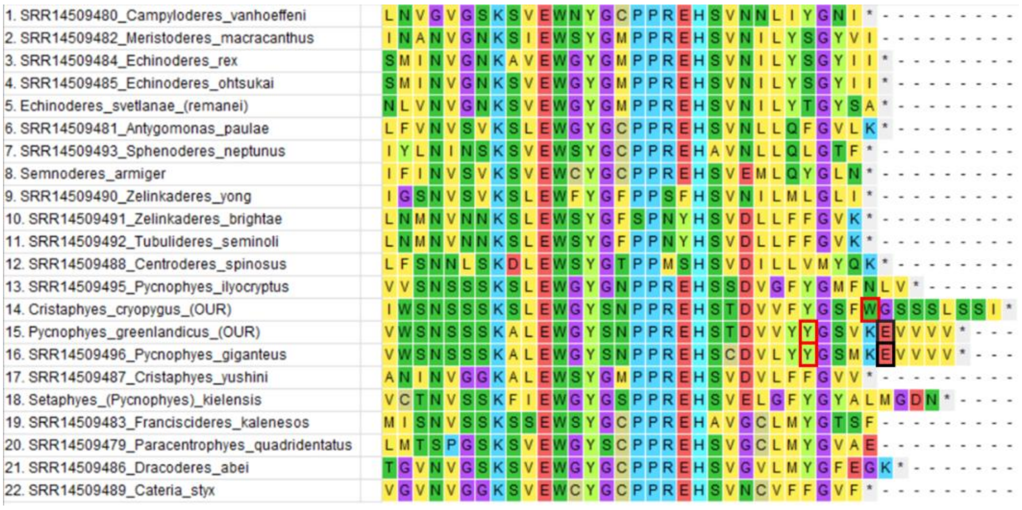

Fig S6. Protein alignment of the COX1 C-terminus (nucleotide 3'end) for 22 known kinorhynch species. Transparent red square boxes indicate potential places for alternative stop codons completed through polyadenylation. Transparent black square boxes indicate codons with the highest polyadenylation signal.
